# Phenotyping of Fecal Microbiota of *Winnie*, a Rodent Model of Spontaneous Chronic Colitis, Reveals Specific Metabolic, Genotoxic, and Pro-inflammatory Properties

**DOI:** 10.1007/s10753-022-01706-0

**Published:** 2022-06-22

**Authors:** Adelfia Talà, Flora Guerra, Silvia Caterina Resta, Matteo Calcagnile, Amilcare Barca, Salvatore Maurizio Tredici, Maria Dolores De Donno, Mirco Vacca, Marina Liso, Marcello Chieppa, Maria De Angelis, Tiziano Verri, Maria Giuseppina Bozzetti, Cecilia Bucci, Pietro Alifano

**Affiliations:** 1 Department of Biological and Environmental Sciences and Technologies, University of Salento, Via Monteroni, 73100 Lecce, Italy; 2 Department of Soil Plant and Food Sciences, University of Bari Aldo Moro, 70126 Bari, Italy; 3 Institute of Research, National Institute of Gastroenterology “S. De Bellis”, 70013 Castellana Grotte (BA), Italy

**Keywords:** *Winnie* intestinal microbiota, phenotyping, inflammation, genotoxicity.

## Abstract

*Winnie*, a mouse carrying a missense mutation in the *MUC2* mucin gene, is a valuable model for inflammatory bowel disease (IBD) with signs and symptoms that have multiple similarities with those observed in patients with ulcerative colitis. MUC2 mucin is present in *Winnie*, but is not firmly compacted in a tight inner layer. Indeed, these mice develop chronic intestinal inflammation due to the primary epithelial defect with signs of mucosal damage, including thickening of muscle and mucosal layers, goblet cell loss, increased intestinal permeability, enhanced susceptibility to luminal inflammation-inducing toxins, and alteration of innervation in the distal colon. In this study, we show that the intestinal environment of the *Winnie* mouse, genetically determined by *MUC2* mutation, selects an intestinal microbial community characterized by specific pro-inflammatory, genotoxic, and metabolic features that could imply a direct involvement in the pathogenesis of chronic intestinal inflammation. We report results obtained by using a variety of in vitro approaches for fecal microbiota functional characterization. These approaches include Caco-2 cell cultures and Caco-2/THP-1 cell co-culture models for evaluation of geno-cytotoxic and pro-inflammatory properties using a panel of 43 marker RNAs assayed by RT-qPCR, and cell-based phenotypic testing for metabolic profiling of the intestinal microbial communities by Biolog EcoPlates. While adding a further step towards understanding the etiopathogenetic mechanisms underlying IBD, the results of this study provide a reliable method for phenotyping gut microbial communities, which can complement their structural characterization by providing novel functional information.

**Supplementary Information:**

The online version contains supplementary material available at 10.1007/s10753-022-01706-0.

## INTRODUCTION

The human gut hosts a complex and dynamically variable microbial community of trillions of microbial cells, which is shaped by host genetics and environmental factors [1]. Over the past 20 years, the number of studies investigating the association between onsets of inflammatory bowel diseases (IBD), comprising ulcerative colitis (UC) and Crohn’s disease (CD), and intestinal microbiota has undergone exponential growth, supported by the availability and large accessibility of ribosomal RNA gene-based metabarcoding and high-throughput metagenomics techniques [2]. While these studies have clearly demonstrated that IBD is associated with dramatic changes in the overall structure of the gut microbiota, specific changes in the gut microbiome and associated inflammatory and metabolic effects on the host in CD and UC are not well-defined [3]. An extensive unbiased meta-analysis of the gut microbiome data from five different IBD patient cohorts from five different countries has recently provided evidence that, while at higher phylogenetic levels gut microbiota community between healthy individuals and IBD patients is highly conserved, at or below the order level in the taxonomic rank, significant disease-specific alterations can be found. In addition, differential enrichment of microbial metabolic pathways in CD and UC, which included enriched pathways related to amino acid and glycan biosynthesis and metabolism, as well as other metabolic pathways, was reported [4].

One of the main obstacles to our understanding of how the gut microbiota affects the health of the host is our scarce information on evolutionary dynamics of the human gut microbiota even in healthy subjects, although its ecological and taxonomic properties have been extensively studied. Recent studies unraveled that the intestinal microbial populations are characterized by extensive longitudinal dynamics, and that, within-host adaptive evolution, these can affect resilience and long-term persistence [5, 6]. These studies open unanswered questions on the role of host-specific factors (such as diet, medication use, and host genetics), and inter-microbial interactions (metabolic interactions, spatial organization, communication signals) in microbial population dynamics. Also, they underlie the need of large longitudinal studies with all relevant information (diet, health status, medication use, weight gain or loss, travel, socioeconomic factors, and host genetics), and approaches for functional and strain-level profiling of the intestinal microbial populations [7]. Moreover, in vitro and in vivo model study systems, such as 3D cell cultures or mouse models, are required to test experimentally, under controlled conditions, working hypotheses mostly deriving from observational studies.

Although more than 60 experimental animal models are currently available for the study of IBD, including chemically induced disease models, congenic mice, models based on immune cell transfer, most of these models induce acute colitis and only a few chronic intestinal inflammations [8]. *Winnie*, a mouse (C57BL/6 background) carrying a missense mutation in the *MUC2* mucin gene, is a valuable model for IBD [9, 10]. In humans, expression of *MUC2* is reduced or depleted in ileal mucosa close to the ulcer margins in CD [11], and MUC2 production is also reduced in active UC [12]. MUC2 mucin, which is the main component of the intestinal mucus being present in the small and large intestine and forming the mucus skeleton, is the best characterized secreted mucin of the gastrointestinal tract [13]. In *Winnie* mice, the intestinal mucus is not firmly compacted in a tight inner layer, becoming permissive for host-microbial interaction [14]. Due to the primary epithelial defect, mice develop chronic intestinal inflammation, with signs of mucosal damage including thickening of muscle and mucosal layers, goblet cell loss, increased intestinal permeability, increased CD45-immunoreactivity in the distal colon, greatly enhanced susceptibility to luminal inflammation-inducing toxins, alteration of innervation in the distal colon, and symptoms of diarrhea, ulcerations, and rectal bleeding similar to those in IBD [14]. Signs and symptoms in *Winnie* mice have multiple similarities with those observed in patients with UC. Mice develop mild and progressive spontaneous inflammation in the distal colon from 6 weeks of age and a severe colitis starts to be detected after 16 weeks of age [15].

The missense mutation in the *MUC2* mucin gene promotes dysbiosis in *Winnie* mice [15]. A recent study compared the gut microbiota of homozygous mutant mice (*Winnie*^−/−^) with their wild-type littermates demonstrating that, although weaned from the same heterozygote mothers (*Winnie*^+/−^) and caged separately according to genotype, dysbiosis in *Winnie* mice was established at 4 weeks of age, before histologic evidence of gut inflammatory changes [15]. Major hallmarks of *Winnie* mice microbiota, as compared with wild-type mice, were greater abundance of Bacteroidetes and Verrucomicrobia, lower abundance of Deferribacteres and Proteobacteria, and, at the species level, a greater abundance of *Akkermansia muciniphila* in 4-week-old mice [15].

In this study, we aimed to answer the question of whether the intestinal environment of *Winnie*, genetically determined by *MUC2* mutation, can select an intestinal microbial community characterized by specific pro-inflammatory properties and metabolic features, which could imply a direct involvement in the pathogenesis of the chronic intestinal inflammation. This question was addressed using a variety of in vitro approaches for gut microbiota functional characterization including cell cultures and co-culture models for evaluation of geno-cytotoxic and pro-inflammatory properties, and cell-based phenotypic testing for metabolic profiling of the intestinal microbial communities.

## MATERIALS AND METHODS

### Mouse Fecal Samples

Stool samples of *Winnie* (WN) and wild-type C57BL/6 (WT) mice 4–16 weeks old were provided by the National Institute of Gastroenterology “S. De Bellis,” Institute of Research, Castellana Grotte, Italy. Fresh stool samples were transported at refrigerated temperature to the Department of Biological and Environmental Sciences and Technologies, Lecce, Italy, and quickly processed for gut microbiota structural and functional characterization.

### Cell Lines and Culture Conditions

Caco-2 and THP-1 cells were grown in RPMI medium 1640 (Gibco) supplemented with 10% fetal bovine serum (FBS) (Gibco), 2 mM L-glutamine, 100 U/mL penicillin, and 10 μg/mL streptomycin. Caco-2 cells were seeded at a density of 1 × 10^5^ cells/mL in 24 multiwell plates (Corning) and allowed to undertake spontaneous differentiation for 21 days with regular medium changes every 5 days. Finally, before the incubations with the fecal material, cells were washed twice with phosphate saline buffer (PBS) and the medium was replaced with RPMI supplemented as above descripted without antibiotics.

For the co-culture system, Caco-2 cells were seeded at a density of 5 × 10^5^ cells/mL in 0.4-µm pore polycarbonate (PC) membrane inserts (Corning) and allowed to differentiate for 21 days with medium changes on days 4, 8, 12, 16, and 18. THP-1 cells were seeded at a density of 3.8 × 10^5^ in 12 multiwell plates (Corning) and subjected to treatment with phorbol 12-myristate 13-acetate (PMA) (2 nM) for 5 days to allow differentiation of THP-1 monocytes into macrophages. Caco-2 and THP-1 cells were washed twice with PBS, the medium was replaced with RPMI supplemented with FBS and glutamine, but without antibiotics. Finally, the membrane inserts were placed in the wells with differentiated THP-1 cells and left overnight before the incubation with the fecal material.

### Confocal Immunofluorescence Analysis

Caco-2 cells grown on membrane inserts were washed in PBS, permeabilized for 2 min with PIPES/EGTA—saponin 0,25% (piperazine-N–N’-bis-(2-ethane sulphonic acid)/ ethylene glycol-bis (β-aminoethyl ether)-N, N, N’, N’-tetraacetic acid-saponin 0,25%), fixed for 20 min with 3% paraformaldehyde in PBS, and washed with PBS. The membrane inserts were cut in two pieces and blocked for 30 min with 3% Bovine Serum Albumin (BSA) (Sigma-Aldrich) and 0,3% Triton X-100 in PBS. For F-actin localization, cells were incubated for 20 min with Actin-Stain 555 Phalloidin (Cytoskeleton) and washed 3 times in PBS. E-cadherin was stained with Anti-E-Cadherin primary antibody (Santa Cruz Biotechnology, sc-21791) diluted 1:400 and, after 3 washes, with Anti-mouse AlexaFluor 488 secondary antibody (Invitrogen) diluted 1:400. Nuclei were labeled with 4’, 6-diamidino-2-phenylindole (DAPI) (1 µg/mL). Membranes were mounted with Mowiol 4–88 (Calbiochem) and viewed with the ZEISS LSM700 (Germany) confocal laser-scanning microscope with a 63 × /1.40 NA oil immersion objective.

### Transepithelial Electric Resistance Measurements

Monolayer integrity of differentiated Caco-2 cells was tested using an epithelial volt-ohm meter (Millicell^®^-ERS, Millipore). The cultures were allowed to equilibrate at room temperature (21–23 °C) for at least 10 min before the transepithelial electric resistance (TEER) measurement in order to reduce any temperature-dependent variability. The TEER was measured twice in each well to reduce technical variability and the mean value was calculated. Finally, the TEER was calculated according to the equation: TEER = (Ω cell monolayer – Ω filter cell-free) × filter area. The final values were expressed as Ω cm^2^.

### Microbial Adhesion Assay

Prior to the adhesion assay, 0.5 g of fresh stool samples of WN and WT mice 4–16 weeks old were resuspended in 5 mL of sterile PBS, and accurately mixed. The suspensions decanted for a few minutes to separate insoluble coarse particles. The resulting homogeneous supernatants were washed three times with PBS and resuspended in antibiotic-free RPMI medium, supplemented with 10% FBS. The suspended biomass concentration was estimated by optical density (OD) measurement and, then, fecal suspensions were added to each well at a multiplicity of infection (MOI) of 100.

Incubation was performed for 3 or 6 h for fluorescence microscopy analysis of bacteria adhering to Caco-2 cells. At each time, the medium was discharged and cells were rinsed three times with PBS to remove non-adherent bacteria. Then, Syto9 (ThermoFisher) green-fluorescent nucleic acid stain was used to visualize microbial cells. For gene expression analyses, the fecal suspensions were incubated for 6 or 9 h with Caco-2 cells, while the incubation with the Caco-2 THP-1 co-culture system was performed for 9 or 12 h.

### Peptidoglycan Detection and Quantification by Immunoblotting

Caco-2 cells, incubated for 6 h with fecal material from WT and WN mice as above described, were washed three times with PBS to remove non-adherent bacteria and resuspended in RIPA (Radioimmunoprecipitation Assay) buffer (50 mM Tris–HCl, pH 8.0, with 150 mM sodium chloride, 1.0% Igepal CA-630 (NP-40), 0.5% sodium deoxycholate, and 0.1% sodium dodecyl sulfate) plus protease inhibitor cocktail (Roche, Mannheim, Germany) and phosphatase inhibitors (phosphatase inhibitor cocktail 1, Sigma-Aldrich). Lysates were incubated for 15 min on ice and subjected to centrifugation at 10,000 rpm for 10 min at 4 °C. The resulting supernatants were transferred into new tubes and used in an immunoblot assay to detect and quantify bacterial peptidoglycan. A Hybri-Slot Manifold (BRL Life Technologies) was assembled with two Whatman 3MM sheets pre-washed in PBS and a PVDF membrane (Amersham™ Hybond™ P 0.45 PVDF) pre-activated in methanol, washed in H_2_O, and equilibrated with PBS. Cell extracts, prepared as above described, were 1:2 serially diluted and a final volume of 200 µL was loaded onto the PVD membrane through the slot apparatus connected to a vacuum pump. Negative and positive controls were also included in the immunoblot assay. Cell extracts of Caco-2 cells not treated with fecal suspension were used as negative control and cell extracts from *Escherichia coli* FB8 (Gram negative) and *Staphylococcus aureus* SA-1 (Gram positive) were used as positive control. The membrane was then blocked with 5% milk in PBS-Tween 0.5% and incubated overnight with mouse anti-peptidoglycan antibody (Bio-Rad, AB620617, 1:1000) and with rabbit anti-histone H3 (Abcam, Ab1791, 1:10,000). Immunoblots were revealed by enhanced chemiluminescence reagent (Bio-Rad, Hercules, California, USA), images were acquired using the ChemiDoc Imaging System (Bio-Rad, Hercules, California, USA), and individual band densities were analyzed with Image Lab™ Software (Bio-Rad, Hercules, California, USA), using histone H3 as normalizer.

### RNA Extraction and Real-Time PCR Analysis

2Total RNA was extracted from Caco-2 and THP-1 cells after incubation with fecal suspension using Aurum™ Total RNA Mini Kit (Biorad) according to the manufacturer’s instructions. Total RNA was quantified using NanoDrop™ (Thermo Scientific, Waltham, MA) and reverse-transcribed using iScript cDNA Synthesis kit (Bio-Rad). Real-time PCR analysis was performed on a CFX96 System (Bio-Rad) using customized 96-well PCR Plates (H96 PrimePCR^®^) (Bio-Rad). H96 PrimePCR^®^ is a pre-optimized assay designed to guarantee high assay specificity, compatibility, avoidance of secondary structures, primer annealing sites and SNPs in target region, and maximized detection of transcript isoforms. Each plate contained lyophilized primers and specific controls for genomic DNA contamination, RNA quality, and efficiency. qPCR data were normalized to the mRNA level of *GAPDH* used as housekeeping gene, and relative mRNA expression was calculated using the ΔΔCt method.

### DNA Extraction from Fecal Material and 16S rRNA Gene-Based Metabarcoding

Total genomic bacterial DNA was extracted from fresh stool samples of 4–16 weeks old *Winnie* mice using the QIAamp DNA Stool Mini Kit (QIAGEN) according to manufacturer’s instructions.

After DNA extraction, DNA library preparation, sequencing, and first-level bioinformatics analysis were carried out by Genomix4life s.r.l. (Baronissi, Salerno, Italy). Quality control and quantification of extracted DNA were performed using NanoDrop ND-1000 spectrophotometer (Thermo Scientific) and Qubit Fluorometer 1.0 (Invitrogen Co., Carlsbad, CA). The hypervariable V3-V4 regions of the 16S rRNA gene were amplified by PCR using the following primers: Forward 5’-CCTACGGGNGGCWGCAG-3’ and Reverse: 5’-GACTACHVGGGTATCTAATCC-3’ [16]. PCR was also performed with a negative control sample, in which no amplification occurred. The libraries were firstly implemented according to 16S Metagenomic Sequencing Library Preparation (Illumina, San Diego, CA) and then quantified. The Phix Control Library (Illumina; expected 25%) was included as a quality control. Before sequencing, each sample was diluted to a final concentration of 4 nM. Finally, samples were subject to cluster generation, and sequenced using MiSeq platform (Illumina, San Diego, CA) in a 2 × 250 paired-end format at a final concentration of 10 pmol.

### 16S rRNA Gene-Based Metabarcoding Data Processing

The quality of the raw sequence files generated by sequencing (fasta files) was assessed using FastQC while chimeric sequences were identified and removed using the UCHIME algorithm (GreenGenes SQL). ClassifyReads, a high-performance implementation of the Ribosomal Database Project (RDP) (http://rdp.cme.msu.edu/), was used to perform taxonomic classification of 16S rRNA gene-targeted amplicons, using the GreenGenes taxonomy database (https://greengenes.secondgenome.com/). This database contains 408,135 quality-filtered complete sequences and is based on a de novo phylogenetic tree obtained using FastTree. The accuracy of the classification algorithm at different taxonomic levels is 100% for kingdom, phylum, and class, 99.98% for order, 99.97% for family, 99.65% for genus, and 98.24% for species. A total of 185,034 raw reads (ranging from 18,622 to 47,147 per sample) were obtained with 100% reads passing quality filtering.

Shannon’s index for WT and WN fecal samples was calculated by Illumina 16S Metagenomics (Version: 1.1.0).

### Genome Prediction of Gene Clusters Coding for Secondary Metabolites

The bacterial version of Antismash web server v 5.0 (default option) was used to identify putative gene clusters coding for secondary metabolism. Genome sequences that were used as input files for AntiSMASH analysis were downloaded from NCBI database. A number of genomes were available in a complete form (chromosome assembly), while other genomes were only available in an uncompleted and unassembled form (i.e., contigs). Table S1 lists the accession numbers of the sequences (chromosome assembly or contig) used to perform this analysis.

### Biolog EcoPlates Inoculation and Incubation

Biolog EcoPlate (BIOLOG Inc., Hayward, CA) is a system of community-level physiological profiling useful to distinguish changes in microbial communities. This method evaluates the metabolic activities of the samples reflecting the functional versatility of microbial communities after the exposition to several stressors. Biolog EcoPlate system is based on a set of 31 carbon substrates and one blank well in triplicate. In detail substrates included 9 carbohydrates (D-Cellobiose, β-Methyl-D-Glucoside, *i*-Erythritol, *N*-Acetyl-D-Glucosamine, Glucose-1-phosphate, α-D-Lactose, D-Xylose, D-Mannitol, D,L-α-Glycerol-phosphate), 10 carboxylic acids and phenols (D-Galactonic acid γ-lactone, γ-Aminobutyric acid, α-Keto butyric acid, Pyruvic acid methyl ester, D-Glucosaminic acid, D-Galacturonic acid, Itaconic acid, D-Malic acid, 2-Hydroxybenzoic acid, 4-Hydroxybenzoic acid), 8 amino acids and amines (L-Arginine, L-Phenylalanine, L-Threonine, L-Asparagine, L-Serine, Glycyl-L-Glutamic acid, Phenylethylamine, Putrescine), and 4 polymers (α-Cyclodextrin, Glycogen, Tween 40, Tween 80). Fecal samples were resuspended and washed twice with 0.85% (w/v) NaCl solution and then diluted to a cell density with an OD_590_ of 0.08. Next, 150 μL of the fecal suspension was inoculated into each well of an EcoPlate. After the inoculum, the EcoPlates were incubated at 37 °C for 1 week in aerobic conditions. In this system, the increase in optical density (OD) at a wavelength of 590 nm values was the indicator of the growth of microbial communities able to use the substrate. We measured the OD every 24 h for each well and the metabolic activity of the overall microbial community was expressed as average well color development (AWCD). AWCD values were calculated by subtraction of the OD values of the blanks from the measured OD values [17].

### Statistical Analyses

Statistical analyses were performed using the PAST 4.03 software. The unpaired two-sample *t*-test was used to determine whether the differences were statistically significant.

## RESULTS

### Structure of the Gut Microbiota from Winnie and C57BL/6 Mice

16S rRNA gene metabarcoding was used to characterize the structure of the bacterial communities from stool samples of WN and WT mice 4–16 weeks old. WT and WN fecal samples showed a similar Shannon diversity index (2.975 and 3.316, respectively).

**Fig. 1:**
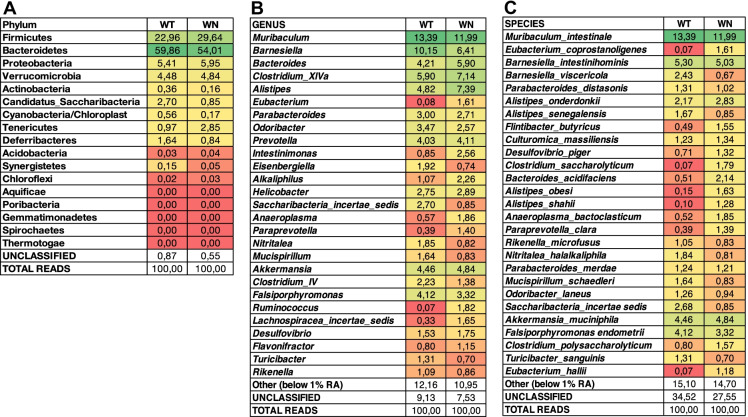
Dominant taxa in *Winnie* (WN) and C57BL/6 (WT) mouse stool samples as determined by 16S rRNA gene metabarcoding. **A**–**C**. Heatmaps show the distribution of the relative abundances of phyla (**A**), and most abundant (> 1%) genera (**B**), and species (**C**).

The 16S Metagenomics (Version: 1.1.0) pipeline was used to determine taxonomic classification. Heat maps show relative abundance of phyla (Fig. 1A) and most represented (> 1%) genera (Fig. 1B) and species (Fig. 1C) in WT and WN samples. The full list of phyla, genera, and species are shown in Tables S2, S3, and S4, respectively. At phylum level, only few differences between the two microbiota could be observed. Firmicutes, Bacteroidetes, Proteobacteria, and Verrucomicrobia were the most abundant phyla, accounting for approximately 92.7% and 94.4% relative abundances in WT and WN, respectively (Fig. 1A). In the WN sample, it was possible to note a lower relative abundance of Bacteroidetes, Actinobacteria, Candidatus Saccharibacteria, Cyanobacteria, Deferribacteres, and Synergistetes, and a greater abundance of Firmicutes, Tenericutes, Acidobacteria, and Chloroflexi compared to WT. Aquificae and Spirochaetes were detected only in WT, while Poribacteria, Gemmatimonadetes, and Thermotogae were only found in WN. Relative abundances of Proteobacteria and Verrucomicrobia were similar in WT and WN.

Greater differences than phylum level could be observed when microbial communities were analyzed at the genus level (Fig. 1B). Eight genera, i.e., *Muribaculum*, *Barnesiella*, *Bacteroides*, *Clostridium* (cluster XIVa), *Alistipes*, *Prevotella*, *Akkermansia*, and *Falsiporphyromonas*, were the most abundant, accounting for 51.07% and 51.09% relative abundances in WT and WN, respectively. Although the cumulative abundance of these genera was similar between the two microbiota (51.07 vs. 51.09%), important differences could be detected in their relative distribution. In the WN sample, it was possible to note a lower relative abundance of *Muribaculum*, *Barnesiella*, *Falsiporphyromonas*, and a greater abundance of *Bacteroides*, *Clostridium* (cluster XIVa), *Alistipes*, and *Akkermansia*. Relative abundances of *Prevotella* were similar in WT and WN.

Among the less abundant genera, *Eubacterium*, *Intestinimonas*, *Alkaliphilus*, *Anaeroplasma*, *Paraprevotella*, *Ruminococcus*, *Lachnospiracea incertae sedis*, and *Flavonifractor* were more represented in WN compared to WT, while *Odoribacter, Eisenbergiella, Saccharibacteria incertae sedis, Nitritalea, Mucispirillum, Clostridium* (group IV)*, Falsiporphyromonas, Turicibacter,* and *Rikenella* were less represented. Relative abundances of *Desulfovibrio* and *Helicobacter* were similar in WT and WN.

These differences at the genus level are reflected at the species level, although caution should be used in the interpretation of the data at the species level due to the inherent limitations of the metabarcoding analysis. Here, the most abundant four species, i.e., *Muribaculum intestinale*, *Akkermansia muciniphila*, *Barnesiella intestinihominis* and *Falsiporphyromonas endometrii*, accounted for approximately 27.26% and 25.19% relative abundances in WT and WN, respectively (Fig. 1C). Relative abundances (within the sample) of these four species were similar in the two microbiomes.

In contrast, marked differences were observed among several less represented species. Specifically, *Eubacterium coprostanoligenes*, *Eubacterium hallii*, *Clostridium polysaccharolyticum*, *Clostridium saccharolyticum*, *Flintibacter butyricus*, *Alistipes onderdonkii*, *Alistipes obesi*, *Alistipes shahii*, *Desulfovibrio piger*, *Bacteroides acidifaciens*, *Anaeroplasma bactoclasticum*, and *Paraprevotella clara* were more abundant in WN compared to WT. In contrast, *Barnesiella viscericola*, *Parabacteroides distasonis*, *Saccharibacteria incertae sedis*, *Turicibacter sanguinis*, *Alistipes senegalensis*, *Rikenella microfusus*, *Nitritalea halalkaliphila*, *Mucispirillum schaedleri*, and *Odoribacter laneus* were less abundant. Relative abundances of *Culturomica massiliensis* and *Parabacteroides merdae* were similar in WT and WN.

It may be interesting to note the different distribution of species within the same genus, namely *Barnesiella intestinihominis* (similar in WT and WN) and *Barnesiella viscericola* (much less abundant in WN); *Parabacteroides merdae* (similar in WT and WN) and *Parabacteroides distasonis* (slightly less abundant in WN); *Alistipes onderdonkii* (slightly more abundant in WN), *Alistipes obesi* and *Alistipes shahii* (much more abundant in WN), and *Alistipes senegalensis* (less abundant in WN).

### Metabolic Profiling of the Gut Microbiota from Winnie and C57BL/6 Mice

**Fig. 2:**
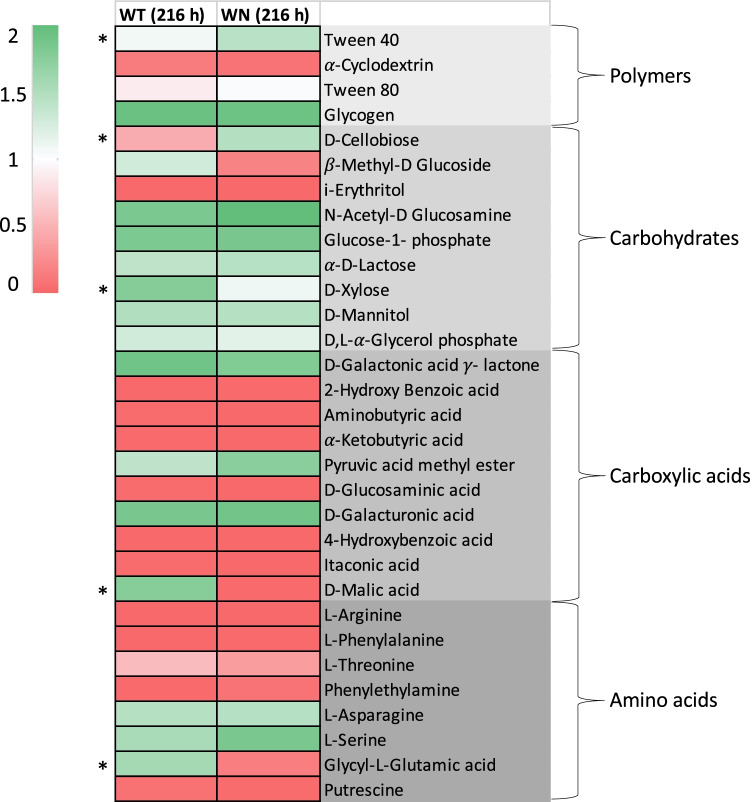
Heatmaps showing Biolog EcoPlate metabolic profiling of *Winnie* (WN) and C57BL/6 (WT) mouse fecal microbial communities. The ability to assimilate carbohydrates, carboxylic acids, amino acids, and polymers was measured at 216 h. All measurements were performed in triplicate for each substrate and statistically significant differences (*p* < 0.05) were marked with an asterisk.

Metabolic patterns of the microbial communities from stool samples of WN and WT were evaluated by BIOLOG system (Figs. 2, S1-S4).

The growth of the microbial communities was analyzed, at different time points (0, 24, 48, 72, 96, 168, 216 h), in the presence of 31 substrates including 9 carbohydrates, 10 carboxylic acids and phenols, 8 amino acids and amines, and 4 polymers, as previously detailed.

Among carbohydrates, WT and WN microbes were able to utilize all substrates, with the exception of the *i*-Erythritol (Figs. 2, S1). However, β-Methyl-D-Glucoside and D-Xylose appeared to be much better assimilated by WT microbes, while D-Cellobiose was better utilized by WN microbes. Among carboxylic acids and phenols, WT and WN microbial communities were able to grow efficiently only on D-Galactonic acid γ-lactone, Pyruvic acid methyl ester, and D-Galacturonic acid, while only the WT microbes were able to utilize D-Malic acid (Figs. 2, S2). When microbial growth was tested in the presence of amino acids and amines, an appreciable growth was detected only in the presence of L-Asparagine, L-Serine, and, to a lesser extent, L-Threonine, while only WT microbes were able to utilize Glycyl-L-Glutamic acid (Figs. 2, S3). Among polymers, α-Cyclodextrin was not assimilated by WT and WN microbial communities; Glycogen, Tween 40, and Tween 80 were utilized by both community without substantial differences (Figs. 2, S4).

**Fig. 3:**
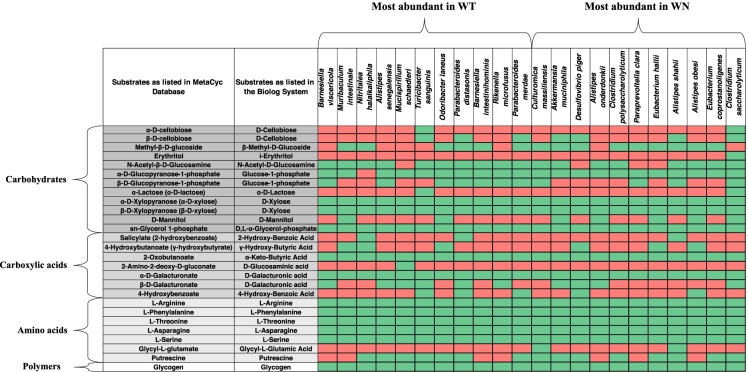
Matching of the substrates utilized by *Winnie* (WN) and wild-type C57BL/6 (WT) mice microbial communities in the Biolog EcoPlates with the substrates that were associated with the most abundant bacterial species (> 1%) in the WT and WN gut microbiota by consulting the MetaCyc Metabolic Pathway Database (https://metacyc.org/). The green box means that substrate is used, and the red box means that substrate is not used by bacterial species. The following species are not listed in the MetaCyc database: *Falsiporphyromonas endometrii***,** *Flintibacter butyricus***,** *Anaeroplasma bactoclasticum* and *Bacteroides acidifaciens.* The following substrates are not used by any of the bacteria listed in MetaCyc database and are not shown in the figure: D-Galactonic Acid γ-Lactone, Pyruvic Acid Methyl Ester, Itaconic Acid, D-Malic Acid, Phenylethylamine, Tween 40, α-cyclodextrin and Tween 80. Aminobutyric acid is not included in the MetaCyc Metabolic Pathway Database.

Overall, these data show the usefulness of the Biolog EcoPlate system in metabolic phenotyping of the gut microbiota, highlighting interesting differences between WT and WN microbes. These were then investigated by matching the substrates utilized by WT and WN microbial communities in the Biolog EcoPlates with the substrates that were associated with the most abundant bacterial species (> 1%) in the WT and WN gut microbiota by the MetaCyc Metabolic Pathway Database (Fig. 3). Results demonstrated a good match for carbohydrate substrates, confirming the potential of the most abundant bacterial species in the WT and WN gut microbiota to assimilate all substrates, except for *i-*Erythritol.

A rather good correspondence was also found for assimilated amino acid and amine substrates (L-Asparagine, L-Serine, and L-Threonine), although MetaCyc revealed an ability to utilize also L-Arginine, L-Phenylalanine, and Putrescine, which was not revealed on Biolog EcoPlates under aerobic incubation conditions. As for carboxylic acids and phenols, MetaCyc confirmed a poor ability to assimilate D-Glucosaminic acid and Itaconic acid (not shown in Fig. 3), and a good ability to assimilate D-Galacturonic acid. MetaCyc revealed the possibility of also using α-Ketobutyric acid, 2-Hydroxybenzoic acid, 4-Hydroxybenzoic acid, which was not revealed on Biolog EcoPlates, although the ability to assimilate 2-Hydroxybenzoic acid and 4-Hydroxybenzoic acid was limited to a small number of species. MetaCyc failed, however, to reveal the utilization of D-Galactonic acid γ-lactone, Pyruvic acid methyl ester, and D-Malic acid.

### Geno-cytotoxic and Pro-inflammatory Properties of the Gut Microbiota from Winnie and C57BL/6 Mice Assayed with Caco-2 Cells

**Fig. 4:**
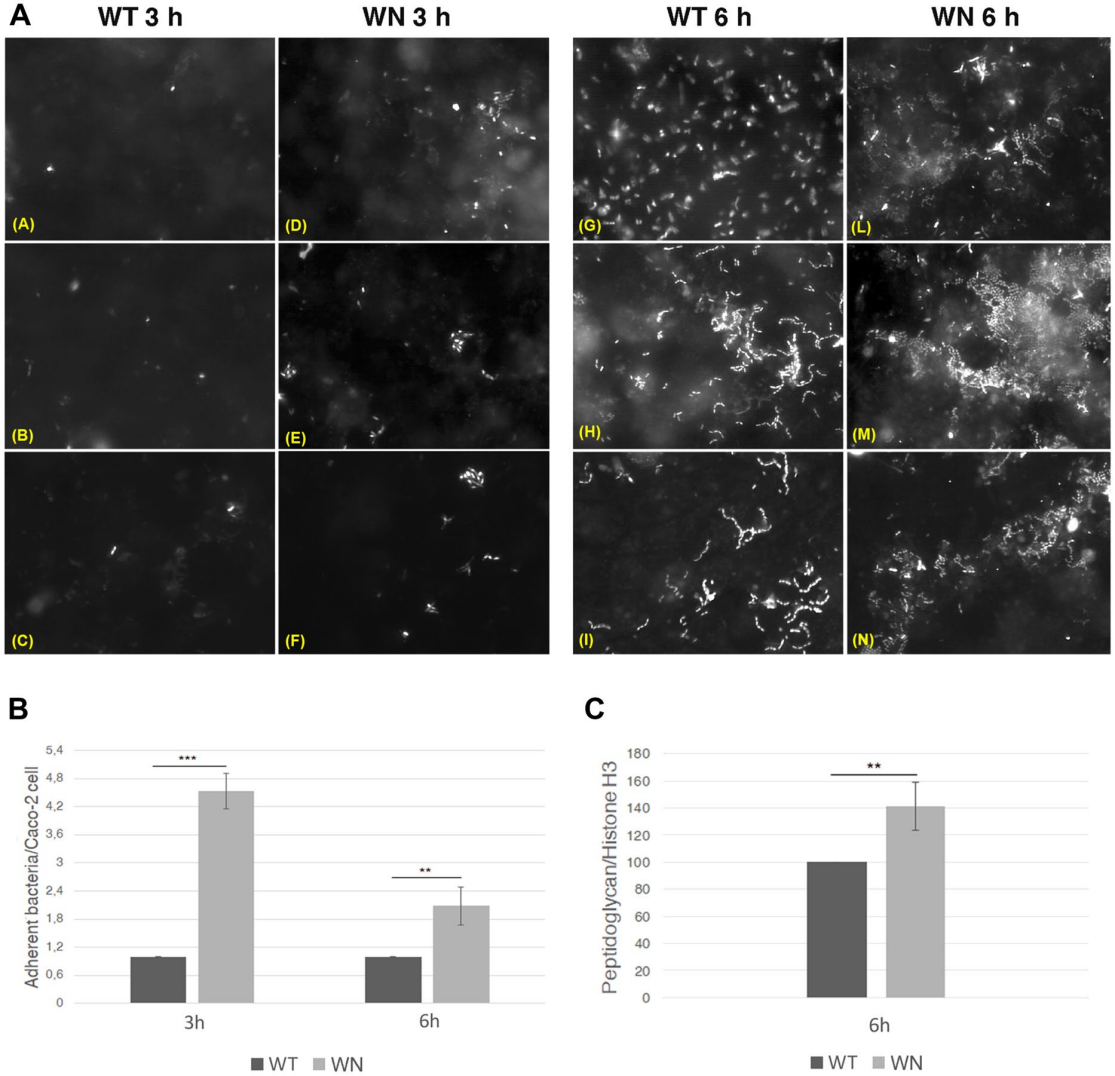
Analysis of fecal bacterial adhesion to Caco-2 cells by fluorescence microscopy and peptidoglycan immunoblotting. A. Caco-2 cells were incubated with *Winnie* (WN) or C57BL/6 (WT) mouse stool samples, and bacterial adhesion was evaluated by fluorescence microscopy using Syto9 after 3 and 6 h. B. Mean numbers with standard deviation of bacteria adhering to Caco-2 cells as revealed by fluorescence microscopy. Values are normalized to WT fecal samples, posed equal to 1. C. Immunoblot analysis of peptidoglycan levels (normalized to total histone H3) in lysates of Caco-2 cells incubated with *Winnie* (WN) or C57BL/6 (WT) mouse stool samples for 6 h. Values were normalized to WT fecal samples, posed equal to 100. The asterisks represent the *p*-value of the statistical test. Two asterisks (**) mean 0.001 < *p* < 0.01. Three asterisks (***) mean *p* < 0.001.

By using customized qPCR arrays (H96 PrimePCR^®^) (Fig. S5), a panel of 43 marker genes was used to evaluate the geno-cytotoxic and pro-inflammatory potential of the gut microbiota from WN and WT mice on differentiated intestinal Caco-2 cells. To this purpose, stool samples from the 4- to 16-week-old mice were decanted to remove coarse debris, and then used to inoculate Caco-2 differentiated cell monolayer, seeded on multi-well plates 21 days before inoculation. Inoculated and non-inoculated cells were incubated and harvested at different time points. Fluorescence microscopy assay showed that, after 3 and 6 h of infection, the mean number of bacteria adhering to Caco-2 cells was about four and two-fold higher, respectively, when cells were incubated with WN stool sample compared to WT stool sample (Fig. 4A, B). To further investigate this evidence, we tried to detect and quantify the bacterial peptidoglycan in WT- and WN-infected cell extracts, as a direct measurement of Caco-2 adherent bacteria (Figs. 4C, S6). Peptidoglycan detection and quantification, performed by immunoblot analysis, revealed that in Caco-2 cell extracts, treated with WN fecal samples, the peptidoglycan level was about 0.4-fold higher than that detected in Caco-2 cell extracts incubated with WT fecal samples (Figs. 4C, S6).

**Fig. 5:**
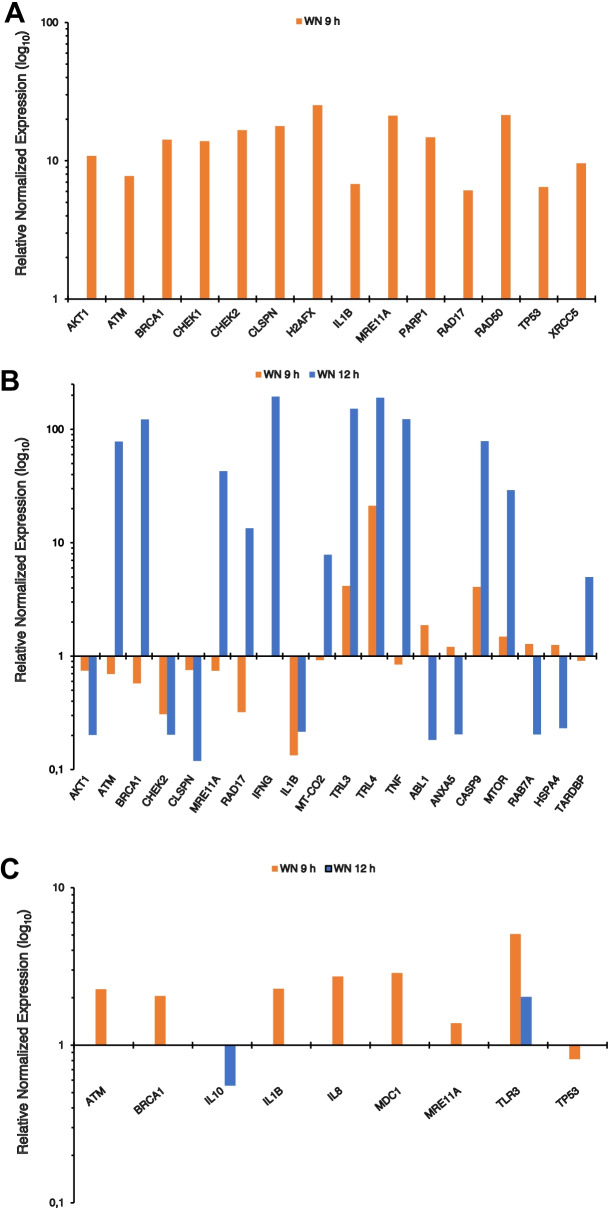
Differential mRNA levels of pro-inflammatory and geno-cytotoxic marker genes in Caco2 cells or Caco2-THP-1 co-cultures incubated with *Winnie* (WN) or C57BL/6 (WT) mouse stool samples as determined by RT-qPCR. **A**. Normalized mRNA levels of pro-inflammatory and geno-cytotoxic marker genes after 9 h of treatment of Caco2 cells seeded on multiwell plates with stool samples from WN or WT mice. **B**, **C**. Normalized mRNA levels of pro-inflammatory and geno-cytotoxic marker genes in Caco-2 cells (**B**) or THP-1 cells (**C**), which were seeded, respectively, on the top and the bottom of the Transwell system, after 9 or 12 h of treatment of with stool samples from WN or WT mice.

The expression of the 43 marker genes was determined 9 h after inoculation, as at that time the pro-inflammatory response could be better assessed and the cells were still viable. Results demonstrated significant upregulation of mRNA levels of 14 genes in the Caco-2 cells after 9 h of exposure to WN stool sample compared to the same cells exposed to WT stool sample. These genes include those coding for RAC-alpha serine/threonine-protein kinase (*AKT1*), breast cancer type 1 susceptibility protein (*BRCA1*), checkpoint kinase 1 (*CHEK1*), checkpoint kinase 2 (*CHEK2*), claspin (*CLSPN*), histone family member X (*H2AFX*), interleukin 1 beta (*IL1B*), double-strand break repair protein MRE11 (*MRE11A*), poly [ADP-ribose] polymerase 1 (*PARP*-1), cell cycle checkpoint protein RAD17, DNA repair protein RAD50, tumor protein P53 (*TP53*), and X-Ray Repair Cross Complementing 5 (*XRCC5*) protein Ku80 (Fig. 5A; Table S5). No significant changes were detected in the expression of the remaining 34 genes tested. It may be seen that most of upregulated genes are involved in cell cycle regulation, and response to DNA damage, in addition to IL1B whose gene product plays a key role in the inflammatory response.

### Geno-cytotoxic and Pro-inflammatory Properties of the Gut Microbiota from Winnie and C57BL/6 Mice Assayed with Caco-2 and PMA-Differentiated THP-1 Co-culture System

To better simulate the gut’s complex microbiome environment, a cell co-culture model was implemented using Caco2 cells and THP-1, a human monocytic cell line. Caco-2 cells were seeded in co-culture with THP-1 cells in the Transwell system, as previously described. To verify cell monolayer integrity, TEER measurement was performed and only wells with values up to 350 Ω cm^2^ were used as this is the threshold proposed for Caco-2 cell line [18]. Moreover, the actin and E-cadherin immunostaining was performed on Caco-2 cells on filters after 9 h of infection. As shown in Fig. S7, the epithelial integrity was conserved also after bacterial infection. Inoculated and non-inoculated cells were incubated and harvested at 9 h and 12 h.

In the co-culture system, where the Caco-2 cells expose only the apical domain to the microbes, the mRNA expression pattern of the 43 selected genes at 9 h in Caco2 cells (Fig. 5B; Table S6) was different compared to that observed with Caco-2 alone at the same time point (Fig. 5A). Indeed, all the genes upregulated in the previous infection did not change their expression in this system, while we observed upregulation of caspase-9 (*CASP9*), Toll-like receptor 3 (*TLR3*) and Toll-like receptor 4 (*TLR4*) mRNAs, and a downregulation of *IL1B* mRNA after exposure to WN stool sample compared to the same cells exposed to WT stool sample. Gene expression was also evaluated after 12 h of exposure to the microbes, and we observed that the gene expression variations observed at 9 h were greatly enhanced in most of cases. Furthermore, compared to the Caco-2 cells exposed to WT stool sample, in the cells exposed to WN stool sample, we observed upregulation of Autofagy Related 7 (*ATG7*), ATM Serine/Threonine Kinase (*ATM*), *BRCA1*, Interferon Gamma (*IFNG*), *MRE11A*, Mitochondrially Encoded Cytochrome C Oxidase II (*MT*-*CO2*), Mechanistic Target Of Rapamycin Kinase (*mTOR*), Nuclear Factor Kappa B Subunit 1 (*NFKB1*), *RAD17*, RB Transcriptional Corepressor 1 (*RB1*), TAR DNA Binding Protein (*TARDBP*) and Tumor Necrosis Factor (*TNF*) mRNAs, and downregulation of ABL Proto-Oncogene 1 (*ABL1*), *AKT1*, Annexin A5 (*ANXA5*), *CHEK2*, *CLSPN* and Ras-Related Protein Rab-7a (*RAB7A*) mRNAs (Fig. 5B).

The same analysis was performed on THP-1 cells stimulated by Caco-2 cells seeded on the filter support on top of THP-1 cells and exposed to the microbes. After 9 h of infection, an upregulation of *TLR3* mRNA was observed compared to control, while the *CHEK2* mRNA could not be detected in THP-1 cells exposed to WN stool sample. After 12 h of infection, the mRNA levels of *TLR3* returned comparable to control. Also, in this case, the *CHEK2* mRNA could not be detected in cells exposed to WN stool sample. Finally, *ATM*, *BRCA1*, *IL1B*, *IL8*, *MDC1*, *MRE11A*, and *TP53* mRNAs were not detected (Fig. 5C; Table S7).

## DISCUSSION

The global burden of IBD, including UC and CD, can be seen in the context of current epidemiological transition [19]. Collectively, these chronic relapsing disorders of the intestinal tract, which can result in a range of debilitating symptoms including abdominal pain, diarrhea, vomiting and rectal bleeding, reduced quality of life, and physical, emotional, social and family problems, affect more than 2 million Europeans and 1.5 million North Americans, and about 6–8 million people are estimated to be affected by IBD worldwide [20]. While in Western countries the incidence of IBD has stabilized, and prevalence is increasing as a result of improved survival, in newly industrialized countries in South America, Eastern Europe, Asia, and Africa, the incidence is rapidly increasing even though the prevalence is still low [21]. The rising incidence in newly industrialized countries parallels with westernization of diet and culture, specifically with an increased intake of processed food, refined sugars, dairy, and less plant-based fibers [22].

Although the exact etiopathological mechanism remains elusive, it is generally accepted that IBD is the result of an inappropriate immune response against environmental factors, including luminal and microbial antigens, in genetically predisposed patients [23]. In the past, a large number of genome-wide association studies has led to the identification of more than 200 polymorphic loci genetically associated with IBD, some of which are also associated with other immune-mediated disorders, most notably with ankylosing spondylitis and psoriasis, and a considerable overlap between susceptibility loci for IBD and mycobacterial infection [24]. Most risk variants are shared across diverse ethnical groups, apart from several major European risk variants, which are not present in eastern Asians, including IL23R, and NOD2 variants that, notably, affect the interaction with the gut bacteria [24]. Despite substantial investments, these genetic studies have not yet brought health benefits through the identification of either genetic risk factors of disease susceptibility or prognostic markers or tools for predicting the response to therapy [25]. However, many studies have highlighted the importance of genetic traits that affect inflammation and the interaction with the intestinal microbiota [25]. This has shifted attention to the gut microbiota as a major player in IBD, the element on which host genetics and the environmental cues actually converge [26].

In this framework, the present study aimed to evaluate if and how a host genetically determined intestinal environment, such as that of the *Winnie* mouse with the *MUC2* mutation, could select an intestinal microbial community characterized by specific pro-inflammatory properties and metabolic features, which may be directly implicated in IBD pathogenesis. It should be noted that in vitro approaches were mostly used in this study, which, due to their reductionistic nature and limitations (including (i) treatment and analysis of the microbiota samples under aerobic conditions; (ii) defined cell infection systems; (iii) limited set of metabolic, geno-cytotoxic, and pro-inflammatory markers), place caveat in the final interpretation of biological relevance and translational relevance of findings. Nonetheless, these approaches have provided some new insights that will certainly need to be confirmed in more appropriate in vivo settings as discussed below.

**Fig. 6:**
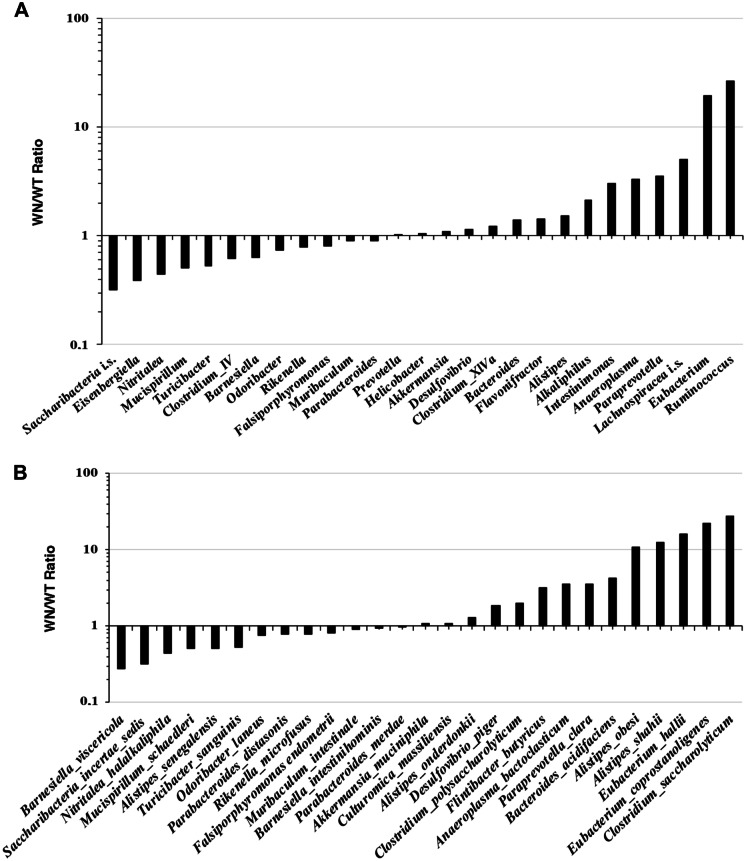
Relative abundances of the dominant taxa in *Winnie* (WN) and C57BL/6 (WT) mouse stool samples as determined by 16S rRNA gene metabarcoding. **A**, **B**. Ratios of relative abundance of most represented bacterial genera (**A**) and species (**B**) in WN and WT stool samples are shown.

We started with 16S rRNA gene metabarcoding analysis that revealed substantial differences in the structure of gut bacterial communities from 4- to 16-week-old WN stool samples compared to parental WT mice (Fig. 1). It is worth to note that, at the genus level and, even more so, at the species level, the greatest differences concerned the less represented taxa (Fig. 6A, B). This situation can be well exemplified by *Alistipes*. We found *Alistipes onderdonkii* slightly more abundant in WN, *Alistipes senegalensis* less abundant in WN, while both *Alistipes obesi* and *Alistipes shahii* were much more abundant in WN. The different abundance of *Alistipes* species in WN and WT samples could be linked to different functional properties of each species in the relationship with the host. *Alistipes* is a relatively new sub-branch genus of the Bacteroidetes phylum with contrasting roles in host–pathogen interaction [27]. *Alistipes* has been involved in the progression of liver disease by affecting bile acids and short-chain fatty acid metabolism, progression of cardiovascular disease and hypertension, modulation of gut inflammation and immune response in cancer, and mental health [27]. On one hand, there is some evidence that *Alistipes* may protect against liver fibrosis, colitis, and cardiovascular disease, as well as against cancer immunotherapy by modulating the tumor microenvironment. On the other hand, several studies suggest that *Alistipes* may be pathogenic in colorectal cancer and may be associated with mental signs of depression [28]. These different behaviors may be due to specific properties of each species, although it is also conceivable that different *Alistipes* species may have different behaviors with respect to host nutrition and health depending on the host and host health. Noteworthy, among the two species much more abundant in WN than in WT, *Alistipes obesi* is the only motile species within the genus *Alistipes*, while *Alistipes shahii* has a clearly defined pro-inflammatory activity by TLR4-priming/TNF production that may increase local inflammation but may also lead to beneficial immunomodulation in cancer treatment [27, 28].

Compared to WT, WN samples were also more enriched in cellulosolytic genera including *Ruminococcus*, *Eubacterium*, and Lachnospiraceae incertae sedis (i.s.) (Fig. 1), consistently with Biolog EcoPlate data showing that D-Cellobiose was better utilized by WN microbes (Fig. 2). *Ruminococcus*, *Eubacterium*, and Lachnospiraceae incertae sedis (i.s.) were, respectively, 25-, 20-, and fivefold more abundant in WN than in WT samples (Fig. 6A). This situation is reflected at the level of species with a prevalence of *Clostridium saccharolyticum*, *Eubacterium coprostanoligenes*, and *Eubacterium hallii* in WN samples (Fig. 6B). The family of Lachnospiraceae and the genus *Eubacterium* include major butyrate-producers capable of utilizing complex carbohydrates such as D-Cellobiose for growth [29, 30]. *Flintibacter butyricus*, which can produce butyrate from carbohydrates, was also enriched in WN samples [31]. Among *Eubacterium* species, it may also be worth to note the ability of *E. coprostanoligenes* to carry out the conversion of cholesterol to the poorly absorbed sterol coprostanol [32]. It is rather surprising to find prevalence in WN mice of cellulolytic and butyrate-producing bacteria, generally associated with a diet based on vegetables rich in fibers and a healthy state of the intestinal microenvironment [33–35]. Wide evidence emphasized that butyrate-producing bacteria establish positive metabolic interactions with the intestinal cells. In fact, butyrate is crucial to maintain a healthy gut as it fuels enterocytes, signals to the host and is a critical mediator of the colonic inflammatory response, and the number of butyrate-producing bacteria is significantly reduced in a variety of diseases, including IBD and diabetes [36].

Apparently, this finding may be in contrast with the geno-cytotoxic and pro-inflammatory potential of WN mouse microbes compared to those of the WT mouse (Fig. 6). Our results suggest that, regardless of the *MUC2* mutation, the *Winnie* gut microbiota seems to be sufficient to elicit an inflammatory response by showing for the first time the genes involved in the aforementioned response in two different in vitro systems. This finding is consistent with a recent study showing that *Winnie* colitis was substantially reduced although not abolished under germ-free conditions [37]. Furthermore, intestinal organoids obtained from *Winnie* mice retain the chronic intestinal inflammatory features characteristic of the parental tissue [38]. Despite the differences with the results obtained with Caco-2 cells seeded on plastic wells, also in the co-culture system, WN stool sample upregulated in Caco-2 cells genes mostly related to DNA damage repair, as compared to WT stool sample. WN microbes also upregulated several genes that are associated with pathogen-associated molecular patterns (PAMPs) and inflammatory response (*TLR3*, *TLR4*, *IFNG*, *NFKB1*, and TNF), while they downregulated genes mostly linked to cell proliferation and differentiation (Fig. 5).

Of note, the intestinal immune system is extremely dynamic, adapting the immune response to mucosal specific factors able to educate the intestinal immune cells [39]. The content of the intestinal lumen, including microbial communities, food-derived antigens, and various metabolites, directly influences the function of innate and adaptive immune cells in a cascade of events finally modulating host immune homeostasis [40]. Intestinal dysfunctions, particularly those involving *TLRs*, *IFNG, NFKB1*, and *TNF*, have established pathogenic roles in several mouse models of colitis and in the onset of human IBD [41, 42]. Lipopolysaccharide (LPS) signaling though TLR4, which requires the accessory molecules CD14, PBP, and MD-2, plays a key role in the pathogenesis of IBD. Under physiological conditions, the expression of this receptor complex is generally low in intestinal cells, but its expression is significantly upregulated by various cell types in both nonactive and active IBD and in experimental mouse models of colitis [43–46]. Upregulation of *MD-2/TLR4* may also result from stimulation of ligands other than LPS, and, furthermore, interferon gamma (IFN-g) and tumor necrosis factor alpha (TNF-a), which play significant roles in triggering IBD, have been found to upregulate intestinal epithelial *TLR4* expression in vitro [47, 48]. Importantly, there is evidence that in active IBD, variant alleles in *TLR4* induce dysregulation, and “gain-of-function” mutations exhibit pro-inflammatory effects in response to physiological LPS concentrations. In particular, D299G polymorphism, which occurs at a frequency of about 6% in Caucasian populations, has been associated with CD and UC [49]. Thus, it has been suggested changes in the microbiota composition in the genetically susceptible host may result in aberrant TLR4 hyperresponsiveness of the intestinal mucosa in IBD patients [48]. Our data appear to be consistent with this hypothesis. Conversely, while there is strong evidence that *TLR4* is significantly upregulated in both UC and CD, *TLR3*, which was upregulated by WN microbes, was shown to be significantly downregulated in intestinal epithelial cells in active CD but not in UC [50]. However, the combination of genetic variations in *TLR3* and *TLR7* has been reported to influence the severity of UC, and TLR3 and TLR4 agonists stimulate IFN-g production [51].

The enhanced geno-cytotoxic and pro-inflammatory potential of the *Winnie* gut microbiota may be related to an enhanced capability to establish physical (and metabolic) interactions with intestinal cells consistently with the results of bacteria-Caco-2 cell adhesion assay (Fig. 4). This may be the consequence of a process of microbial selection triggered by the alteration of the intestinal micro-environment caused by the missense mutation in the *MUC2* gene. MUC2 mucin is present in *Winnie* but is not firmly compacted in a tight inner layer [10]. It is reasonable to assume that the lower compactness of intestinal mucin in the *Winnie’s* gut can favor the contact of microbes with the epithelial surface and, therefore, locally favor the selection of groups of microorganisms that interact closely with the intestinal cells. This pathogenetic hypothesis, which is focused on the role of two major players, i.e., the intestinal mucin metabolism and the local microbiota, in inducing or enhancing the inflammatory state through a self-sustaining process, could be tested in vivo by fecal microbiota transplant experiments (FMT) with germ-free or antibiotic-treated mice [52–54]. If the biological relevance of our findings were confirmed with FMT experiments in mice, it would open up the possibility of extending these findings to human IBD.

There is strong evidence that implicates mucin biosynthesis and turnover in IBD pathogenesis. In IBD cases, structural changes in the glycoprotein nucleus of mucin, sulfation, and sialylation of mucin oligosaccharide residues and quantitative changes in mucin production are commonly observed [55]. These changes, which lead to a diminished functionality of the mucous barrier, have been associated to various genetic mutations or polymorphisms, but they may also be induced by exposure to environmental cues [56–58]. Dynamic changes in mucin glycosylation pattern may be induced by the local microbiota, immune factors, and dietary components (fat, fiber, prebiotics, protein, and food additives) [59]. For instance, there is evidence that dietary fiber appears to improve intestinal barrier function in pigs through increased mucin production capacity (i.e., goblet cell numbers, *MUC2* gene expression) and secretion (i.e., fecal mucin output) [60]. Mucin synthesis and mucin degradation are in equilibrium in healthy individuals. There is accumulating evidence that in UC patients, there is a decrease in the number of goblet cells and subsequently a decrease in mucin secretion, and an increase in the relative number of immature goblet cells that produce unstable hypo-glycosylated and/or hypo-sulfated mucin [55]. This results in infiltration of the luminal microbiota into the inner mucus layer, and increased contact with intestinal cells leading to inflammation. Our results with the *Winnie* mouse model, if confirmed by FMT experiments in mice, would allow us to add another piece to this picture: an increased contact between microbes and the epithelial surface (as a consequence of less compact and partially de-structured *MUC2* mucin) may, in turn, favor the proliferation in the gut of bacteria whose lifestyle is determined by intimate interactions with the intestinal cells. The pro-inflammatory properties of these microbes may amplify and self-sustain the inflammatory process.

Were the biological relevance of our findings confirmed in vivo, it would also open up the possibility of using the proposed in vitro approaches for gut microbiota phenotyping in the clinical practice. Indeed, the gut microbiome exhibits high taxonomic compositional variation across individuals and extensive temporal dynamics [61], although gene composition and functional capabilities tend to be conserved. Indeed, there the gut microbiota is characterized by high degree of functional redundancy since phylogenetically unrelated taxa may carry out similar functions [62]. Moreover, remarkable functional variations exist even within the same species [63]. These limitations make it difficult to draw firm conclusions from the structural analysis of the microbiome using conventional rRNA-based methods, and highlight the need to develop new strategies and approaches, which can be suitably transferred to clinical practice. High-throughput metagenome sequencing, metatrascriptomics, single-cell sequencing, proteomics, and metabolomics are all very powerful technologies, but difficult to apply in clinical routine. Better applicability might have several methods that aim to comprehensively assess the phenotype of a microbial community such as those proposed in this study.

Our phenotypic profiling also shows more pronounced ability of WN microbes, as compared to WT microbes, to induce the expression of a number of DNA damage repair related genes in Caco-2 cells (Fig. 5). Thus, it would be interesting to understand if the bacteria that are numerically enriched in the WN sample have the capacity to produce genotoxic substances. It is well known that patients with IBD, including CD and UC, have an increased risk of developing colorectal cancer, and have, in their gut, higher levels of bacteria producing colibactin, a genotoxic peptide-polyketide hybrid molecule produced primarily by some *E. coli* strains [64]. This group of strains may also encode cyclomodulins, cycle inhibiting factors (CIF) capable of blocking mitosis independently of DNA damage and inducing apoptosis in exposed epithelial cell lines [65, 66]. Moreover, in colorectal cancer cases driven by chronic inflammation, mutations in TP53 gene are found to be an early event [67]. In addition to colibactin-positive and CIF-positive *E. coli*, other bacteria with potential genotoxic capacity have been associated with IBD, including several strains of *Bacteroides fragilis* producing metalloproteinase enterotoxin (BFT), and *Enterococcus faecalis* producing genotoxic peroxide [68]. Genotoxicity of BFT is the result of cross-talk with intestinal cells; it has been associated to the ability of BFT to induce an intense Th-17-mediated inflammatory response [69], but also to induce cleavage of E-cadherin, leading to increased paracellular permeability and the activation of β-Catenin and a subsequent increase in cell proliferation [70]. Other by- or end-products of microbial primary metabolism, such as H_2_S produced by sulfate-oxidizing bacteria, and phenol, indoles and 4-cresol produced by bacteria that ferment aromatic amino acids, could also contribute to genotoxicity [68]. Finally, we must not forget the ability of some bacteria to produce secondary metabolites with powerful genotoxic capabilities, such as, for example, the enediynes which are produced by certain groups of actinomycetes, and which are used in the clinic as anticancer agents [71]. In this regard, it may be interesting to note greater abundance of gene clusters encoding secondary metabolites in the bacterial species that were enriched in WN compared to WT mice (Tables 1 and 2). Genome analysis of reference strains belonging to these species (Table S1) by antiSMASH revealed a substantial enrichment for cluster encoding arylpolyenes, linear azol(in)-e-containing peptides, non-ribosomal peptides, thiopeptides, ranthipeptides and RiPP recognition element (RRE) for peptide maturation in bacteria coming from *Winnie* mouse (Tables 1 and 2). Therefore, if this picture is correct, manipulation of the gut microbiota could represent an important prophylactic or therapeutic strategy in controlling IBD progression and colorectal cancer.

**Table 1:** Secondary Metabolite Gene Clusters Identified by AntiSMASH in Genomes of Reference Strains of Prevalent Species in WN or WT Mice

**Bacteria**	**Sample**	**RRE**^**a**^	**Thiopeptide**	**Ranthipeptide**	**NRPS**^**b**^	**Arylpolyene**	**PKS**^**c**^	**LAPs**^**d**^	**Resorcinol**	**CDPS**^**e**^	**Terpene**	**Lanthipeptide**	**β-lactone**	**RiPP**^**f**^
***Akkermansia muciniphila***	WN	**-**	**-**	**-**	**-**	** + **	**-**	**-**	**-**	**-**	** + **	**-**	**-**	**-**
***Alistipes onderdonkii***		** + **	**-**	**-**	**-**	** + **	**-**	**-**	**-**	**-**	**-**	**-**	**-**	**-**
***Desulfovibrio piger***		** + **	** + **	**-**	**-**	**-**	** + **	**-**	**-**	**-**	**-**	**-**	**-**	**-**
***Clostridium saccharolyticum***		**-**	**-**	**-**	**-**	**-**	**-**	**-**	**-**	**-**	**-**	**-**	**-**	**-**
***Alistipes obesi***		**-**	**-**	** + **	**-**	**-**	**-**	**-**	**-**	**-**	**-**	**-**	**-**	**-**
***Alistipes shahii***		** + **	**-**	**-**	**-**	**-**	**-**	**-**	**-**	**-**	**-**	** + **	**-**	**-**
***Eubacterium coprostanoligenes***		** + **	**-**	** + **	**-**	**-**	**-**	**-**	**-**	**-**	**-**	**-**	**-**	**-**
***Anaeroplasma bactoclasticum***		** + **	**-**	**-**	** + **	**-**	**-**	**-**	**-**	**-**	**-**	**-**	**-**	**-**
***Culturomica massiliensis***		**-**	**-**	**-**	** + **	**-**	**-**	**-**	**-**	**-**	**-**	**-**	** + **	**-**
***Clostridium polysaccharolyticum***		**-**	**-**	** + **	** + **	**-**	** + **	** + **	**-**	**-**	**-**	**-**	**-**	** + **
***Bacteroides acidifaciens***		** + **	**-**	**-**	**-**	**-**	**-**	**-**	**-**	**-**	**-**	**-**	**-**	**-**
***Paraprevotella clara***		** + **	**-**	**-**	**-**	** + **	**-**	**-**	** + **	**-**	**-**	**-**	**-**	**-**
***Eubacterium hallii***		**-**	**-**	** + **	** + **	**-**	**-**	**-**	**-**	**-**	**-**	**-**	**-**	**-**
***Barnesiella viscericola***	WT	**-**	**-**	**-**	**-**	**-**	**-**	**-**	**-**	**-**	**-**	**-**	**-**	**-**
***Barnesiella intestinihominis***		**-**	**-**	**-**	**-**	**-**	**-**	**-**	**-**	**-**	**-**	**-**	**-**	**-**
***Rikenella microfusus***		**-**	**-**	**-**	**-**	**-**	**-**	**-**	**-**	**-**	**-**	**-**	**-**	**-**
***Alistipes senegalensis***		**-**	**-**	**-**	**-**	**-**	**-**	**-**	**-**	**-**	**-**	**-**	**-**	**-**
***Mucispirillum schaedleri***		**-**	**-**	**-**	**-**	**-**	**-**	**-**	**-**	**-**	**-**	**-**	**-**	**-**
***Muribaculum intestinale***		**-**	**-**	**-**	**-**	** + **	**-**	**-**	**-**	**-**	**-**	**-**	**-**	**-**
***Parabacteroides distasonis***		**-**	**-**	**-**	**-**	**-**	**-**	**-**	** + **	** + **	** + **	**-**	**-**	**-**
***Turicibacter sanguinis***		** + **	**-**	**-**	**-**	**-**	**-**	**-**	**-**	**-**	**-**	**-**	**-**	**-**
***Parabacteroides merdae***		**-**	**-**	**-**	**-**	**-**	**-**	**-**	** + **	**-**	**-**	**-**	**-**	**-**
***Odoribacter laneus***		**-**	**-**	**-**	**-**	**-**	**-**	**-**	** + **	**-**	**-**	** + **	** + **	**-**
***Nitritalea halalkaliphila***		**-**	**-**	**-**	**-**	** + **	** + **	**-**	**-**	**-**	**-**	**-**	**-**	** + **

^a^RiPP recognition element (RRE) for peptide maturation

^b^nonribosomal peptide synthases

^c^polyketide synthase

^d^linear azol(in)e-containing peptides

^e^cyclic dipeptides

^f^ribosomally synthesized and post-translationally modified peptides

**Table 2:**
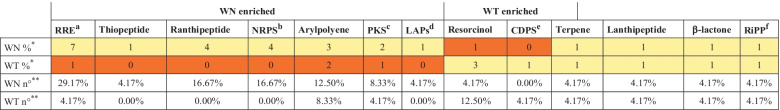
Enrichment Analysis of Secondary Metabolite Gene Clusters in the Bacterial Species of Prevalent Species in WN or WT Mice as Shown in Table 1

^a^RiPP recognition element (RRE) for peptide maturation

^b^non ribosomal peptide synthases

^c^polyketide synthase

^d^linear azol(in)e-containing peptides

^e^cyclic dipeptides

^f^ribosomally synthesized and post-translationally modified peptides

## CONCLUSIONS

In this study, we have pursued two aims: (i) to propose a polyphasic approach for functional characterization of gut microbiota by using in vitro approaches, and (ii) to investigate the role of intestinal microbiota in the pathophysiology of IBD in a *Winnie* mouse model harboring *MUC2* mutation. Firstly, we have characterized the structure of prokaryotic communities in WT and WN fecal microbiota by 16S rRNA gene metabarcoding. Then, the prokaryotic structural profiles were compared with the microbial metabolic profiles of WT and WN stool samples, outlined by Biolog EcoPlates phenotyping. Results emerging from structural analysis were consistent with metabolic data, demonstrating that Biolog EcoPlate technology is a reliable method to explore the metabolic potential of microbiota in animal fecal samples. Afterwards, in order to investigate the ability of fecal bacteria to interact with intestinal cell, a microbial adhesion assay was performed, using Caco-2 cell lines. Two different approaches were used to detect and quantify the number of bacteria adhering to Caco-2 cells, and both revealed that *Winnie* fecal microbiota had greater ability to interact with intestinal cells than WT. The increased capability of *Winnie* fecal microbiota to establish physical and metabolic interactions with intestinal cells compared to WT microbiota was accompanied by enhanced genotoxic, cytotoxic and pro-inflammatory potentials, as determined in two different in vitro cellular systems that were used to screen the mRNA expression pattern of 43 marker genes, by customized qPCR arrays. In conclusion, we both provide (i) a reliable method for phenotyping fecal microbial communities and (ii) in vitro evidence that the *Winnie* mouse fecal microbiota, selected by host genetic alterations, may elicits specific pro-inflammatory, genotoxic, and cytotoxic responses in the intestinal tract, suggesting a direct role of intestinal microbial community in IBD pathogenesis.

## Supplementary Information

Below is the link to the electronic supplementary material.

Supplementary file1 (DOCX 3908 KB)

Supplementary file2 (XLSX 71 KB)

## Data Availability

All data generated or analyzed during this study are included in this published article (and its supplementary information files).
