# Supplementary material for: Phenotyping of Fecal Microbiota of Winnie, a Rodent Model of Spontaneous Chronic Colitis, Reveals Specific Metabolic, Genotoxic, and Pro-inflammatory Properties
Source: Inflammation. 2022 Jun 22;45(6):2477–97. doi: 10.1007/s10753-022-01706-0 (PMC9646540; doi:10.1007/s10753-022-01706-0)
Supplement: Supplementary file 1 — Supplementary file1 (DOCX 3908 KB) [file 10753_2022_1706_MOESM1_ESM.docx]

**SUPPLEMENTARY INFORMATION**

**Phenotyping of fecal microbiota of Winnie, a rodent model of spontaneous chronic colitis, reveals specific metabolic, genotoxic, and pro-inflammatory properties**

Adelfia Talà^1^#, Flora Guerra^1^#, Silvia Caterina Resta^1^#, Matteo Calcagnile^1^, Amilcare Barca^1^, Salvatore Maurizio Tredici^1^, Maria Dolores De Donno^1^, Mirco Vacca^2^, Marina Liso^3^, Marcello Chieppa^3^, Maria De Angelis^2^, Tiziano Verri^1^, Maria Giuseppina Bozzetti^1^, Cecilia Bucci^1^, Pietro Alifano^1^*

**Fig. S1** Biolog EcoPlate metabolic profiling of *Winnie* (WN) and C57BL/6 (WT) mouse intestinal microbial communities. The ability to assimilate carbohydrates was determined at different time points (0, 24, 48, 72, 96, 168 and 216 h). Each measurement has been performed in triplicate and it was shown as mean ± standard deviation.

**Fig. S2** Biolog EcoPlate metabolic profiling of *Winnie* (WN) and C57BL/6 (WT) mouse fecal microbial communities. The ability to assimilate carboxylic acids and phenols was determined at different time points (0, 24, 48, 72, 96, 168 and 216 h). Each measurement has been performed in triplicate and it was shown as mean ± standard deviation.

**Fig. S3** Biolog EcoPlate metabolic profiling of *Winnie* (WN) and C57BL/6 (WT) mouse fecal microbial communities. The ability to assimilate amino acids and amines was determined at different time points (0, 24, 48, 72, 96, 168 and 216 h). Each measurement has been performed in triplicate and it was shown as mean ± standard deviation.

**Fig. S4** Biolog EcoPlate metabolic profiling of *Winnie* (WN) and C57BL/6 (WT) mouse fecal microbial communities. Ability to assimilate polymers was determined at different time points (0, 24, 48, 72, 96, 168 and 216 h). Each measurement has been performed in triplicate and it was shown as mean ± standard deviation.


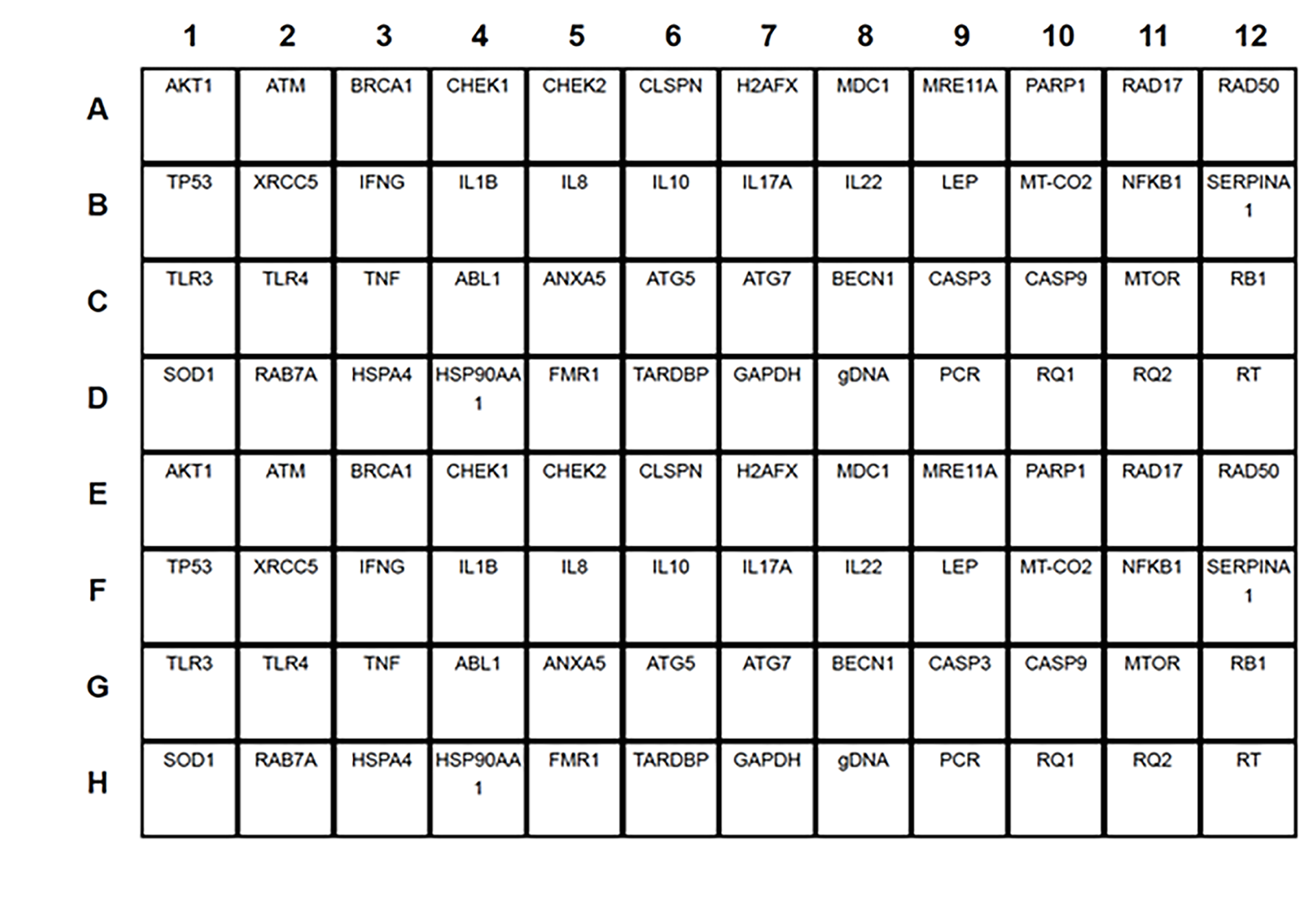


**Fig. S5** Custom 96-well plate for RT-qPCR array (H96 PrimePCR®). Well contains oligonucleotide primers for qPCR analysis of specific mRNAs that are used as markers for genotoxic response (wells: A1 to B2), pro-inflammatory response (wells: B3 to C3), DNA damage response and cytotoxicity (wells: C4 to D6). From D7 to D12 wells: oligonucleotide primers for control gene mRNAs. From E1 to H12: duplicated controls.


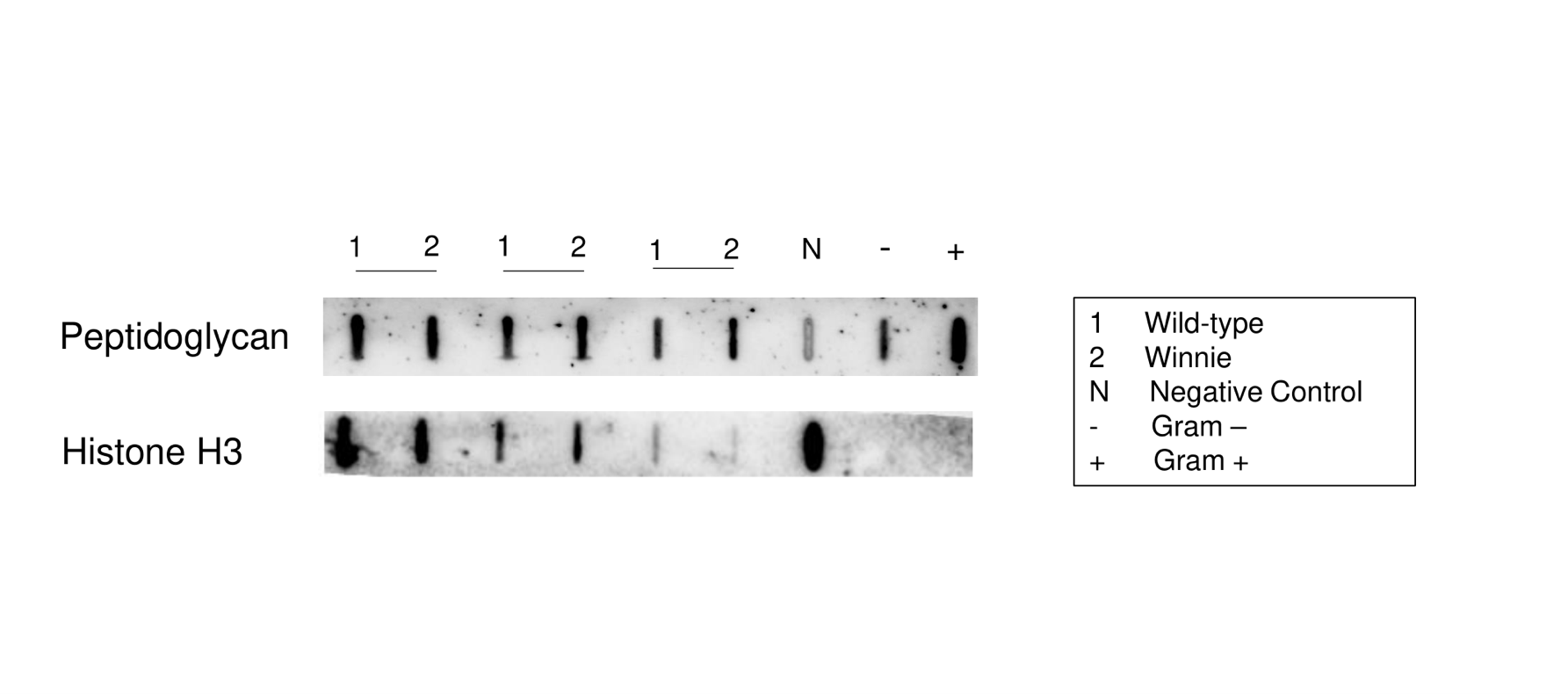


**Fig. S6** Peptidoglycan and Histone H3 immunoblot on Caco-2 cell extract, 1:2 serially diluted, after 6 h infection with stool sample from C57/BL6 (WT) [1] and *Winnie* (WN) [2]. As controls we included cell extract from Caco-2 cells non infected (negative control) [N], a Gram-negative bacterium (*E. coli* FB8) [-] cell extract and a Gram-positive bacterium (*S. aureus* SA-1) [+] cell extract. To analyze the data, background signal from negative control was subtracted. Histone H3 was used as normalizer and difference between samples [1] and [2] was calculated.


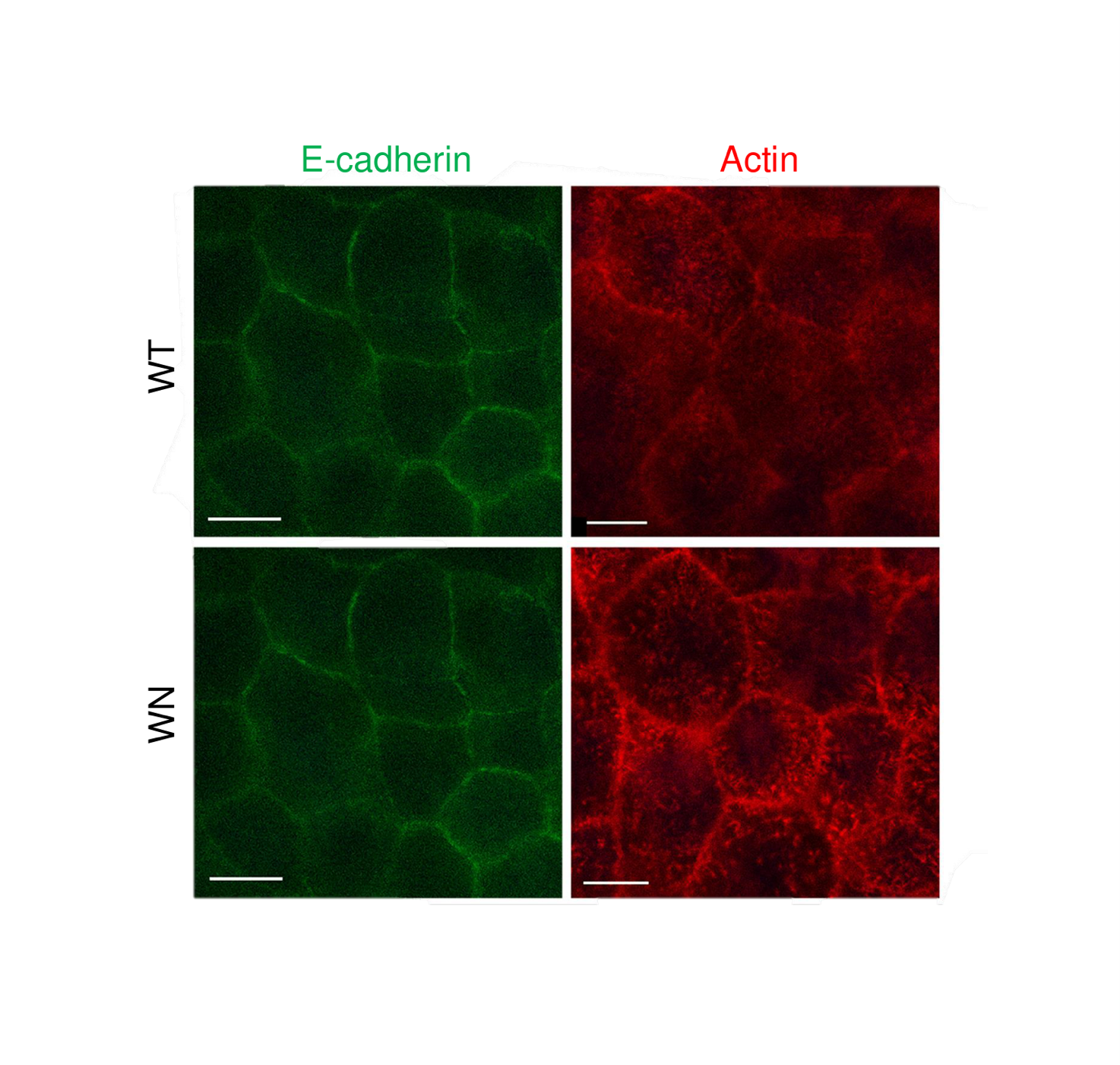


**Fig. S7** Actin and E-cadherin staining. Caco-2 cells seeded on filter support were immuno-stained with antibodies against actin and E-cadherin after cell differentiation and incubation with *Winnie* (WN) or C57BL/6 (WT) stool samples for 9 h. Green labeled E-cadherin is shown in the left panels, while red labeled Actin is shown in the right panels. Scale bar: 10 μm.

**Table S1** Genome assemblies or contigs used to predict secondary metabolite gene clusters by antiSMASH.

| **Bacteria** | **Complete genome** | **Assembly (Contig)** |
| --- | --- | --- |
| ***Akkermansia muciniphila*** | CP001071.1; AP021898.1; CP015409.2 | - |
| ***Alistipes obesi (Alistipes communis)*** | AP019735.1 | - |
| ***Alistipes onderdonkii*** | AP019738.1; AP019737.1; AP019734.1 | - |
| ***Alistipes senegalensis*** | - | UYXI01000001-UYXI01000003 |
| ***Alistipes shahii*** | FP929032.1 | - |
| ***Anaeroplasma bactoclasticum*** | - | QXEV01000001-QXEV01000050 |
| ***Bacteroides acidifaciens*** | - | CAEUHO010000001-CAEUHO010000011 |
| ***Barnesiella intestinihominis*** | - | JADNJV010000001-JADNJV010000011 |
| ***Barnesiella viscericola*** | CP007034.1 | - |
| ***Clostridium polysaccharolyticum*** | - | FOHN01000001-FOHN01000054 |
| ***Clostridium saccharolyticum*** | CP002109.1 | - |
| ***Culturomica massiliensis*** | - | CABKWI010000001-CABKWI010000011 |
| ***Desulfovibrio piger*** | LT630450.1 | - |
| ***Eubacterium coprostanoligenes*** | - | FUWW01000001-FUWW01000036 |
| ***Eubacterium hallii (Anaerobutyricum hallii)*** | CP073280.1 | - |
| ***Mucispirillum schaedleri*** | - | FTRD01000001-FTRD01000036 |
| ***Muribaculum intestinale*** | CP021421.1; CP015402.1; CP065316.1 | - |
| ***Nitritalea halalkaliphila*** | - | AJYA01000001-AJYA01000140 |
| ***Odoribacter laneus*** | - | JADMWH010000001-JADMWH010000033 |
| ***Parabacteroides distasonis*** | CP072254.1; AP019729.1; CP042285.1 | - |
| ***Parabacteroides merdae*** | CP082229.1 | - |
| ***Paraprevotella clara*** | - | JAGZSK010000001-JAGZSK010000028 |
| ***Rikenella microfusus*** | - | ATXW01000001-ATXW01000003 |
| ***Turicibacter sanguinis*** | CP053187.1 | - |

**Legends of Supplementary Tables in Excel files**

**Table S2** Bacterial phyla full list.

**Table S3** Bacterial genera full list.

**Table S4** Bacterial species full list.

**Table S5** ∆∆Ct values and relative normalized mRNA levels of pro-inflammatory and geno-cytotoxic marker genes after 9 h of treatment of Caco2 cells seeded on multiwell plates with stool samples from *Winnie* (WN) or wild type C57BL/6 (WT) mice.

**Table S6** ∆∆Ct values and relative normalized mRNA levels of pro-inflammatory and geno-cytotoxic marker genes in Caco-2 cells, which were seeded, respectively, on the top of the Transwell system, after 9 or 12 h of treatment with stool samples from WN or WT mice.

**Table S7** ∆∆Ct values and relative normalized mRNA levels of pro-inflammatory and geno-cytotoxic marker genes in THP-1 cells, which were seeded, respectively, at the bottom of the Transwell system, after 9 or 12 h of treatment with stool samples from WN or WT mice.
